# Supplementary material for: Histone deacetylase 11 inhibition promotes breast cancer metastasis from lymph nodes
Source: Nat Commun. 2019 Sep 13;10:4192. doi: 10.1038/s41467-019-12222-5 (PMC6744422; doi:10.1038/s41467-019-12222-5)
Supplement: Supplementary file 1 — Supplementary Information [file 41467_2019_12222_MOESM1_ESM.pdf]

Histone deacetylase 11 inhibition promotes breast cancer metastasis from lymph nodes

Patrick L. Leslie, Yvonne L. Chao, Yi-Hsuan Tsai, Subrata K. Ghosh, Alessandro Porrello, Amanda E.D. Van Swearingen, Emily B. Harrison, Brian C. Cooley, Joel S. Parker, Lisa Carey, Chad V. Pecot

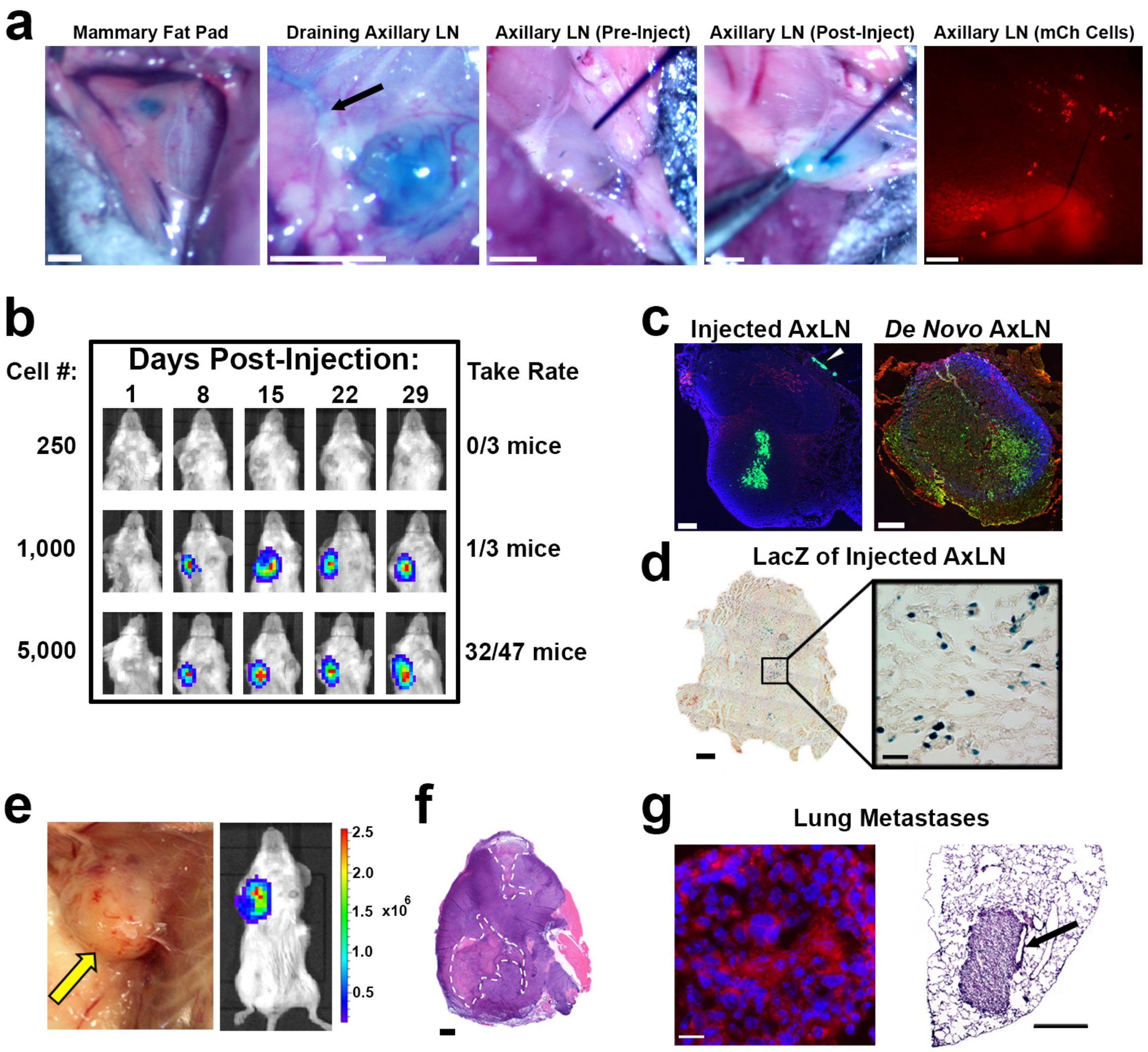

Supplementary Figure 1. Optimization of the LN micro-injection model. a, Patent Blue V was injected into the 8th MFP (first image from left), which can be observed in the draining lymphatics (indicated by an arrow in the 2nd image) to the sentinel axillary lymph node. The third and fourth photo show an axillary LN before and after micro-injection. 4T1 mCh/rL cells can be detected in the axillary LN after injection (fifth image). **b,** Take-rates of 4T1 mCh/rL cells are detected in AxLN post-injection by IVIS imaging. **c,** Micro-injected and de novo AxLN mets are detected by fluorescence imaging. **d,** Micro-injected lacZ-expressing 4T1 cells are detected in the AxLNs after X-gal exposure. **e,** Gross appearance of AxLN tumors 6 weeks post-injection and corresponding IVIS imaging. **f**, H&E staining of micro-injected AxLNs 6 weeks post-injection. Dashed lines indicate regions of necrosis. **g**, De novo lung metastases are observed by fluorescence microscopy and H&E staining 6 weeks after AxLN micro-injection.

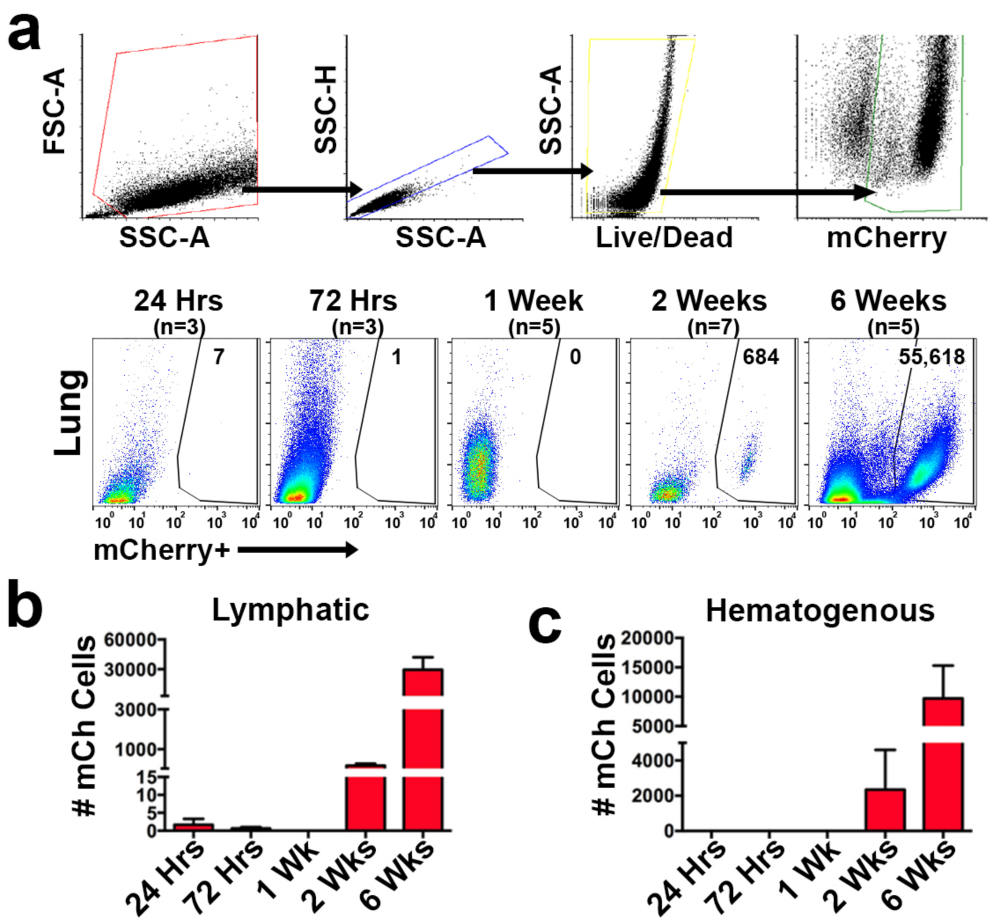

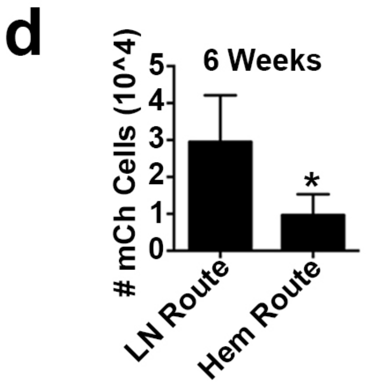

e

Lung Mets by Route (%)

	2 Weeks	6 Weeks
Lymphatic	71%	100%
Hematogenous	33%	67%
P (χ2)	0.04	0.07

f

Brain Mets by Route (%)

	2 Weeks	6 Weeks
Lymphatic	0%	60%
Hematogenous	6%	18%
P (χ2)	0.26	0.03

Supplementary Figure 2. Kinetics of distant metastasis formation after AxLN micro-injection. a, Representative FACS gating strategy (top) and plots (bottom) to detect the number of mCherry (mCh)-positive cells at the indicated time points for the lungs following AxLN micro-injection (n=3-7 for each time point as denoted above panels). **b-d**, LN-microinjected and TV-injected 4T1 mCh/rLuc cells (n=5,000 cells/mouse by each route) were compared for lung metastasis efficacy. Statistical significance was measured by unpaired one-sided students t-test. **e**+**f**, Statistical analysis comparing lung (**a**) and brain metastasis efficiency at 2- and 6-weeks post-injection for LN-microinjected and tail vein-injected mice. Statistical significance determined by chi-squared contingency test. p-values are indicated as * = $p<0.05$

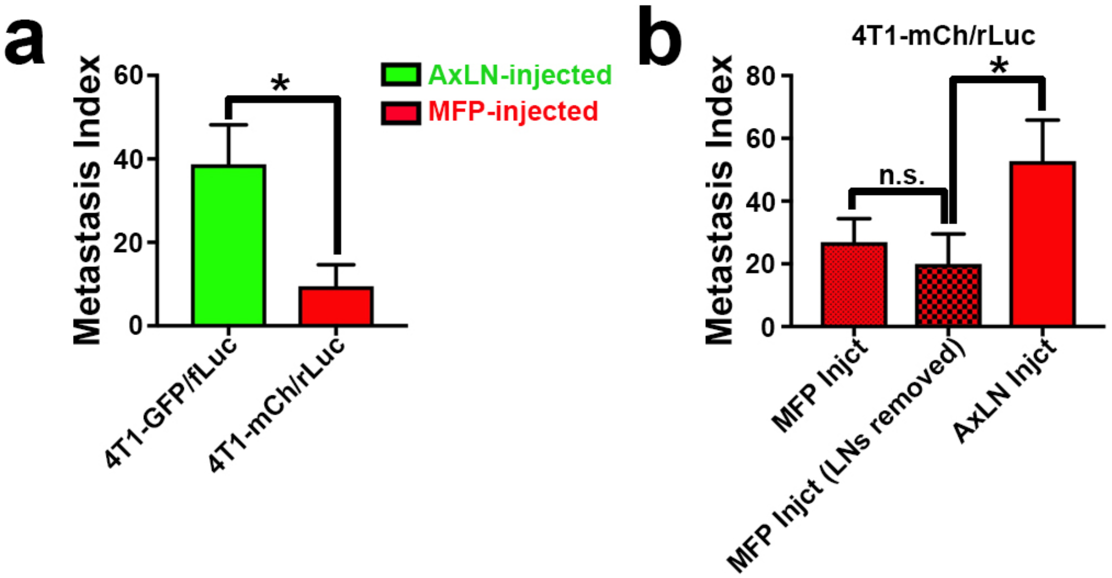

Supplementary Figure 3. Relative distant metastasis by injection route. a, Distant lung metastases were determined by FACS analysis by swapping the reporter cell lines. Equal numbers of cell lines ($5x10^3$) were injected into their respective microenvironment, 4T1-GFP/fL lines were micro-injected into the axillary LN and 4T1-mCh/rL lines were injected into the mammary fat pad (MFP) of the same mouse (n=4 mice). **b**, Comparison of distant lung metastasis by FACS analysis when 4T1-mCh/rL lines were injected into the MFP in mice with intact axillary LNs, into MFPs after axillary LNs were resected, or following micro-injection into the axillary LNs (n=8-10 mice/group). In both experiments, all read-outs were determined at the same time when tumors in any group reached ~1.5 cm in greatest dimension. Statistical significance was measured by an unpaired one-sided student's t-test (**a**) and (**b**) Mann-Whitney test; * = $p<0.05$.

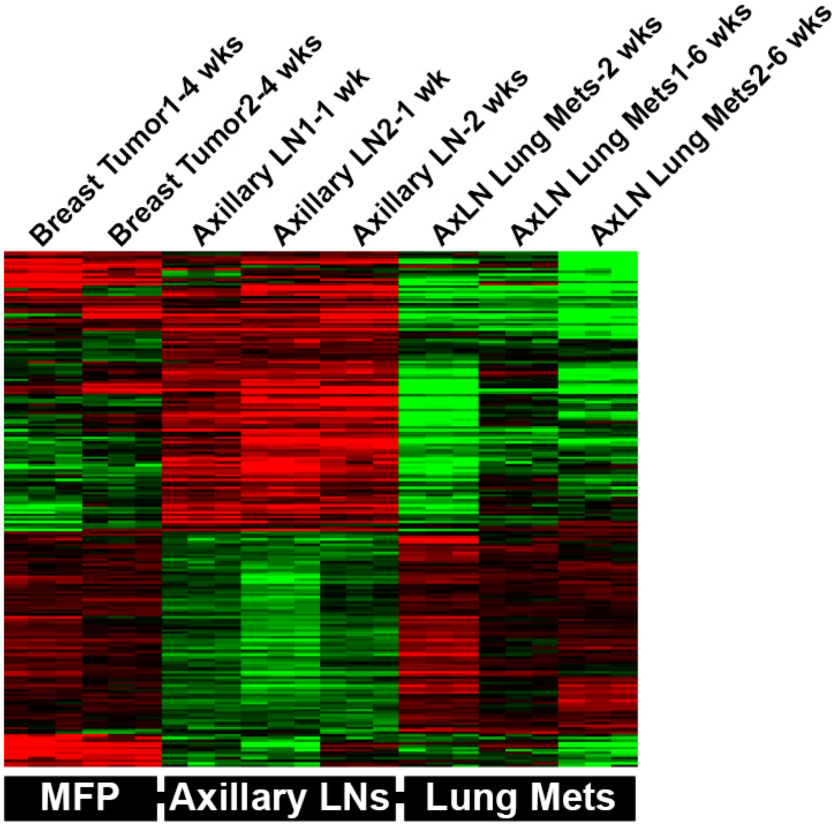

Supplementary Figure 4. Microarray analysis of 4T1 sub-clones from the mammary fat pad, axillary lymph nodes (AxLN) and AxLN-derived lung metastasis. Composite microarray results for the 206 genes that are up- or down-regulated in both pairwise tissue comparisons (refer to Figure 2c+d).

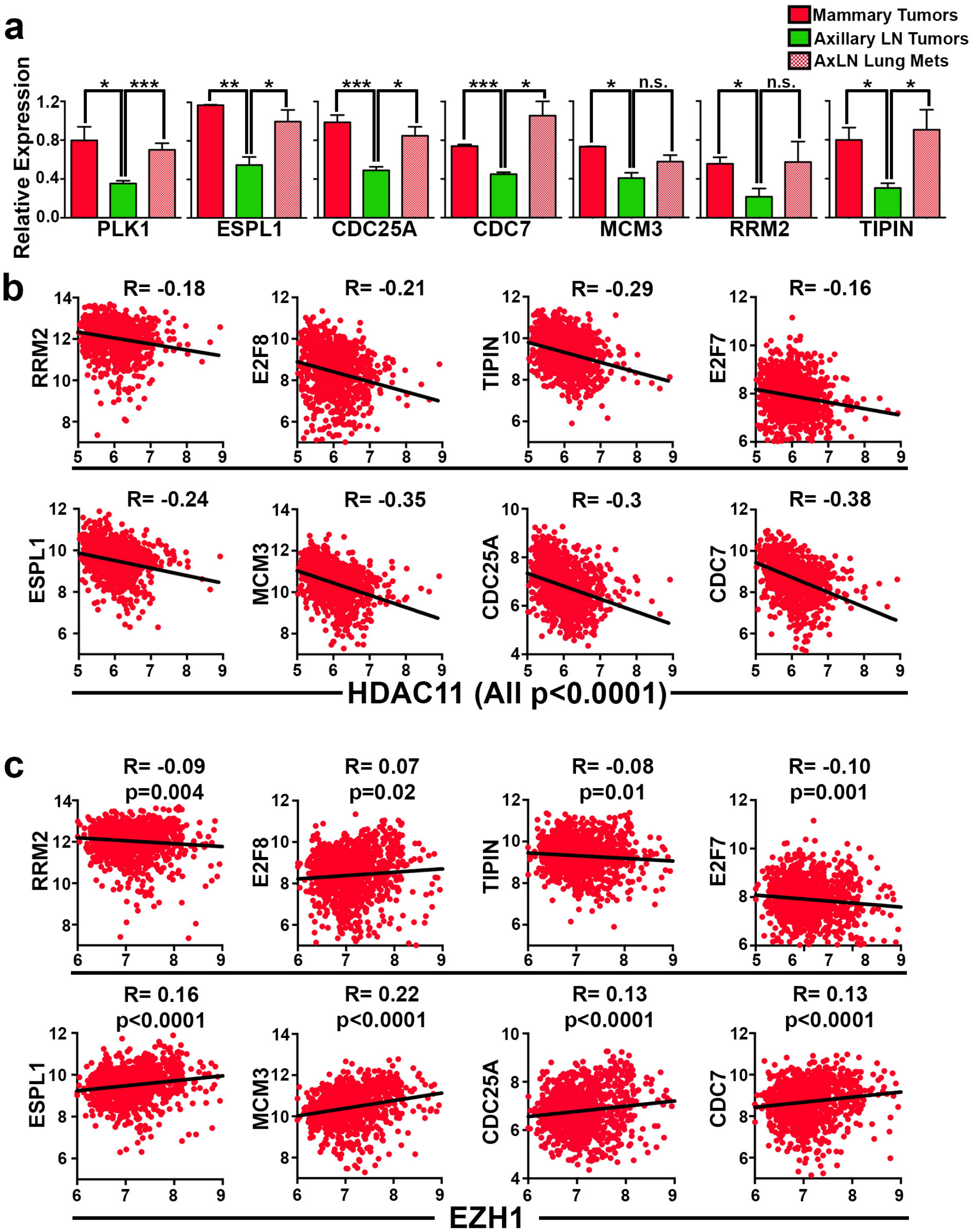

Supplementary Figure 5. HDAC11 expression negatively correlates with the expression of several down-regulated AxLN genes. a, RT-qPCR validation of target gene expression in MFP, AxLN, and lung met sub-clones. Statistical significance was measured by unpaired students t-tests. Cancer cell line encyclopedia analysis of HDAC11 **b**, and EZH1 **c**, expression levels relative to several down-regulated AxLN genes. Pearson's correlation was used to determine statistical significance. p-values are indicated as * = p<0.05, ** = p<0.01, *** = p<0.001.

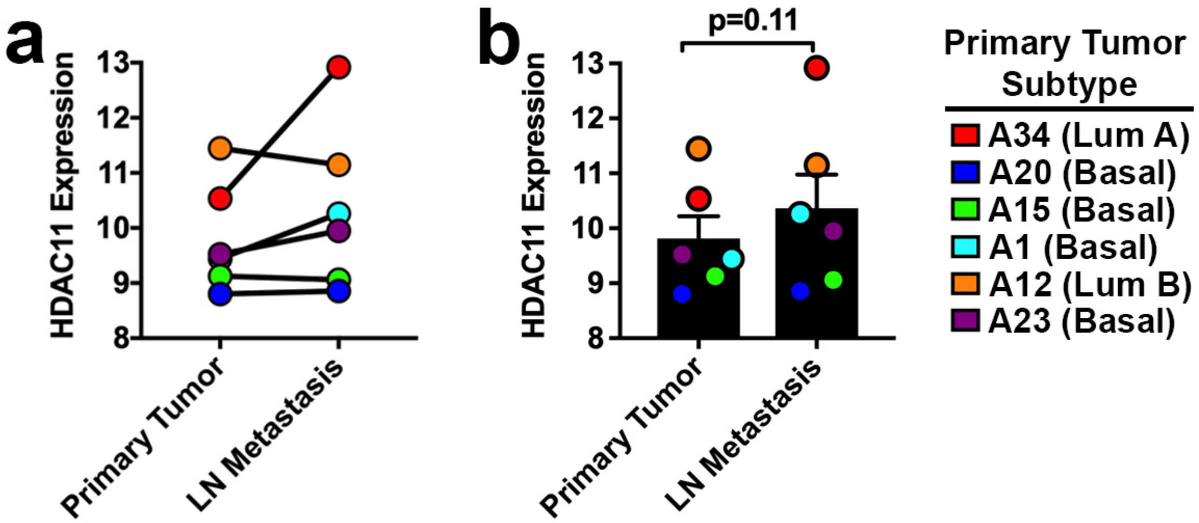

Supplementary Figure 6. HDAC11 expression patterns in matched breast cancer samples from the Rapid Autopsy Program. a, HDAC11 expression patterns by RNAseq of matched primary and LN metastasis obtained from the Rapid Autopsy Program. **b**, Average expression levels of HDAC11 between primary tumors and LN metastasis. Statistical significance was measured by a paired one-sided students t-test.

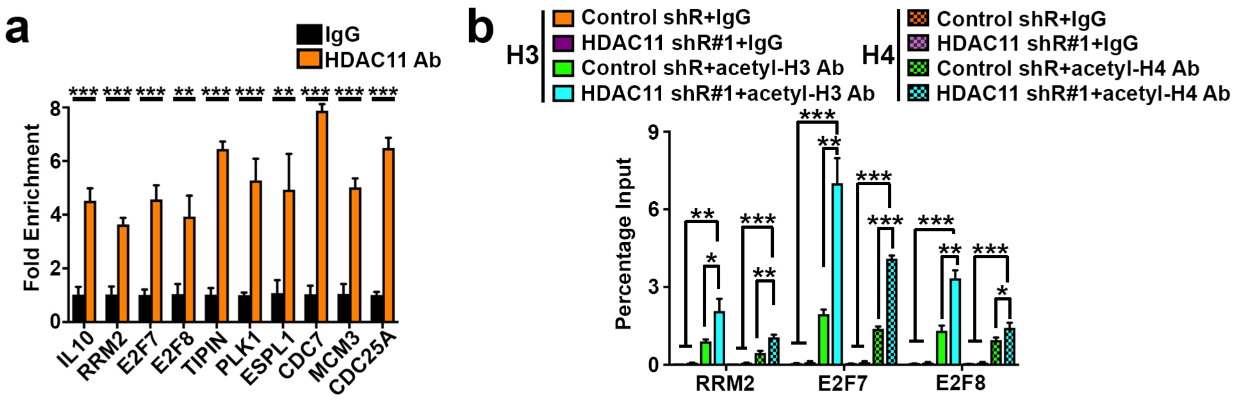

Supplementary Figure 7. Chromatin immunoprecipitation of HDAC11 and acetylated H3 and H4. a, Levels of immunoprecipitated FLAG-HDAC11 in the promoter region of a set of down-regulated array genes in 293T cell lines. Statistical significance was measured by students two-sided t-tests. **b**, Levels of immunoprecipitated promoter regions for RRM2, E2F7 and E2F8 in E0771.LMB cell lines stably expressing either control or HDAC11 shR when pulling down with either IgG control, acetyl-H3 or acetyl-H4 antibodies. Statistical significance was measured by ANOVA. * = p<0.05, ** = p<0.01, *** = p<0.001.

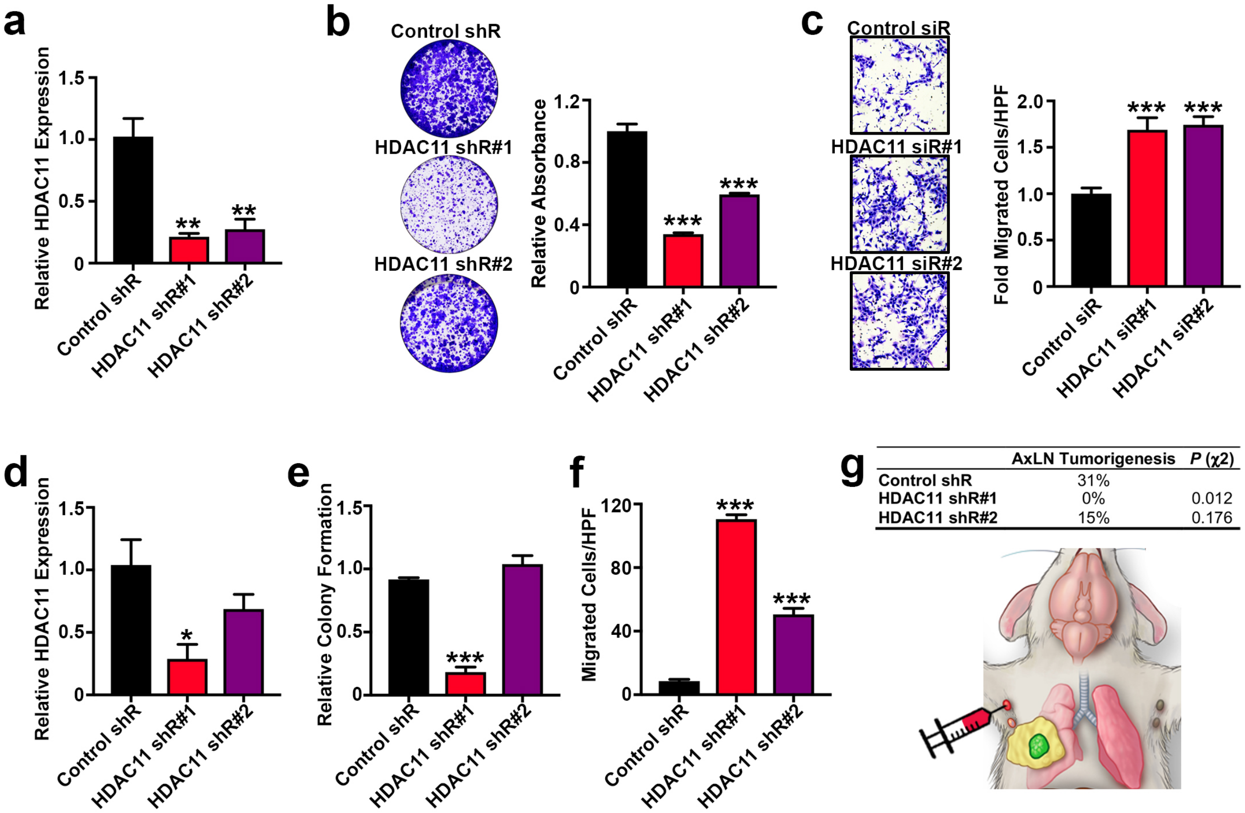

Supplementary Figure 8. HDAC11 silencing blocks colony formation and tumorigenicity but increases migration. **a**, Relative expression of HDAC11 in 4T1-mCh/rL cells stably expressing control shRNA or two different HDAC11 shRNAs. **b**, Representative images of colony formation assays for 4T1 cells subjected to treatment with HDAC11 shRNAs. The graph shows the quantitation of the crystal violet dye. **c**, Representative images and quantification of a transwell migration assay for 4T1 cells following transient knock-down of HDAC11. **d**, Relative expression of HDAC11 in E0771.LMB cells stably expressing control shRNA or two different HDAC11 shRNAs. **e**, Relative colony formation and **f**, transwell migration of E0771.LMB cell lines. **g**, Take-rates of LN-microinjected E0771.LMB shHDAC11 cell lines compared to shCtrl cells (n=13-14 mice/group). P-value obtained using a chi-squared contingency test. Statistical significance (**a-f**) was measured by unpaired two-sided students t-tests; * = p<0.05, ** = p<0.01, *** = p<0.001.

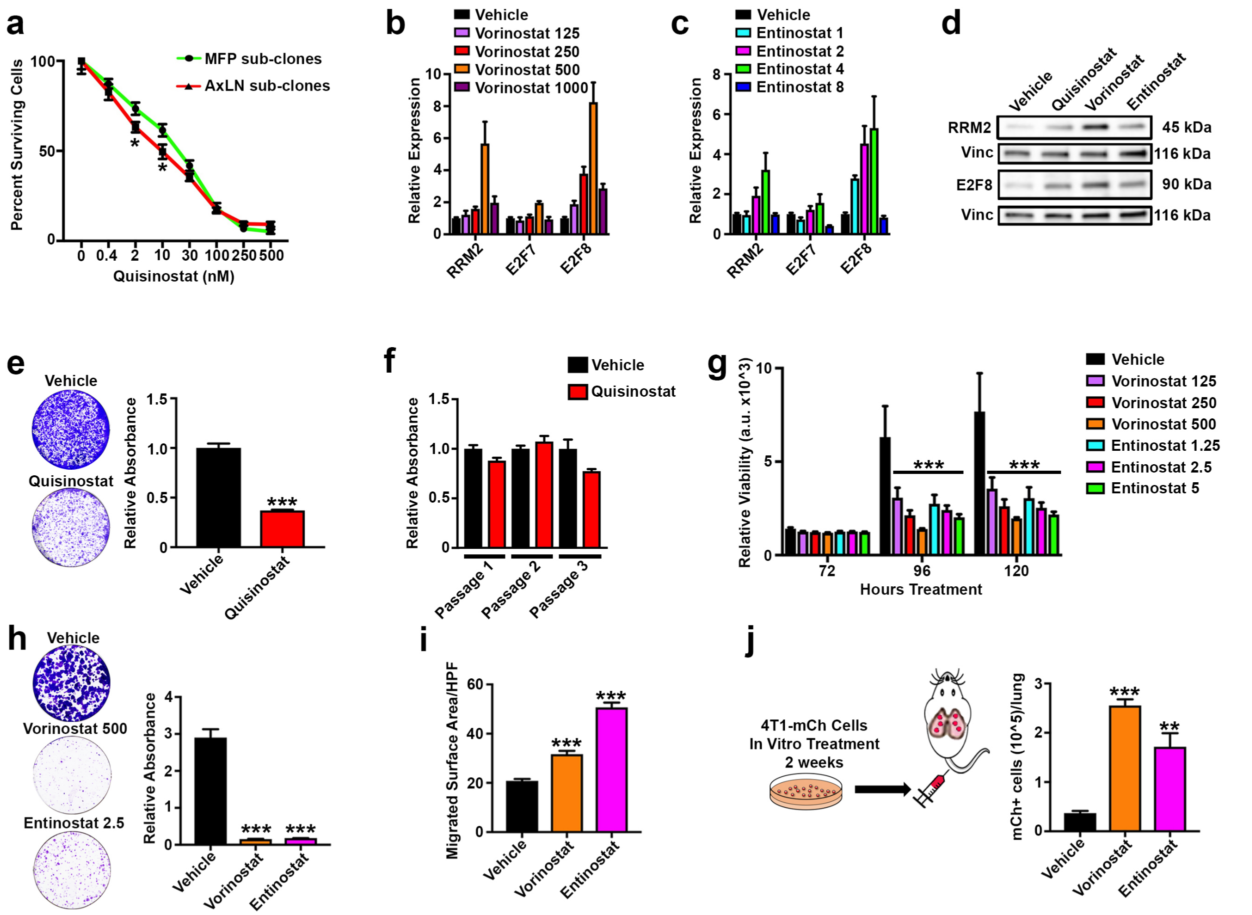

Supplementary Figure 9. HDAC inhibitors block cancer cell growth but promote migration and metastasis. a, Alamar Blue cell growth assay for 4T1 ex vivo MFP and AxLN sub-clones treated with the indicated concentrations of quisinostat. **b**, RT-qPCR analysis of HDAC11 target genes in 4T1 cells treated with the indicated concentrations of vorinostat, or **c**, entinostat. **d**, Western blots for RRM2 and E2F8 following 4T1 treatment with vehicle versus several HDAC-inhibitors. **e**, Representative colony formation images of vehicle versus quisinostat-treated 4T1 cells. The graph to the right shows the quantification of crystal violet stain. **f**, Quantification of crystal violet stain for colony formation results from 4T1 cells that were treated with quisinostat, and then drug was withdrawn before plating for three successive passages post-drug withdrawal. **g**, Alamar Blue cell growth assay for 4T1 cells treated with the indicated concentrations of vorinostat and entinostat at the indicated time points. **h**, Representative colony formation assay results for 4T1 cells treated with 500 nM vorinostat or 2.5 μM entinostat. The graph to the right shows the quantification of the crystal violet stain. **i**, Quantification of migrated cells by transwell migration assay after treatment of 4T1 cells with vorinostat (500 nM) or entinostat (2 μM). **j**) Schematic of TV injection of in vitro-pre-treated 4T1 cells with 500 nM vorinostat or 2 μM entinostat. The graph to the right shows the flow cytometry-based quantification of the mCh-positive cells observed per mouse lung, n= 4-5 mice/group. Statistical significance was measured by unpaired two-sided students t-tests; p-values are indicated as * = p<0.05, ** = p<0.01, *** = p<0.001.
