# Supplementary material for: Histone deacetylase 11 inhibition promotes breast cancer metastasis from lymph nodes
Source: Nat Commun. 2019 Sep 13;10:4192. doi: 10.1038/s41467-019-12222-5 (PMC6744422; doi:10.1038/s41467-019-12222-5)
Supplement: Supplementary file 3 — Reporting Summary [file 41467_2019_12222_MOESM3_ESM.pdf]

nature research

Corresponding author(s): Chad V. Pecot

Last updated by author(s): Aug 23, 2019

Reporting Summary

Nature Research wishes to improve the reproducibility of the work that we publish. This form provides structure for consistency and transparency in reporting. For further information on Nature Research policies, see Authors & Referees and the Editorial Policy Checklist.

Statistics

For all statistical analyses, confirm that the following items are present in the figure legend, table legend, main text, or Methods section.

n/a	Confirmed	
☐	☒	The exact sample size (*n*) for each experimental group/condition, given as a discrete number and unit of measurement
☐	☒	A statement on whether measurements were taken from distinct samples or whether the same sample was measured repeatedly
☐	☒	The statistical test(s) used AND whether they are one- or two-sided<br>*Only common tests should be described solely by name; describe more complex techniques in the Methods section.*
☒	☐	A description of all covariates tested
☐	☒	A description of any assumptions or corrections, such as tests of normality and adjustment for multiple comparisons
☐	☒	A full description of the statistical parameters including central tendency (e.g. means) or other basic estimates (e.g. regression coefficient) AND variation (e.g. standard deviation) or associated estimates of uncertainty (e.g. confidence intervals)
☐	☒	For null hypothesis testing, the test statistic (e.g. *F*, *t*, *r*) with confidence intervals, effect sizes, degrees of freedom and *P* value noted<br>*Give P values as exact values whenever suitable.*
☒	☐	For Bayesian analysis, information on the choice of priors and Markov chain Monte Carlo settings
☒	☐	For hierarchical and complex designs, identification of the appropriate level for tests and full reporting of outcomes
☐	☒	Estimates of effect sizes (e.g. Cohen's *d*, Pearson's *r*), indicating how they were calculated

Our web collection on statistics for biologists contains articles on many of the points above.

Software and code

Policy information about availability of computer code

Data collection	For detailed descriptions for data collection, please refer to methods sections "Rapid autopsy analysis" and "microarray analysis".
Data analysis	For detailed descriptions for data analysis, please refer to methods sections "Rapid autopsy analysis" and "microarray analysis". In brief, the following software packages were used for these analyses: R software package “ape”, using all detected somatic mutations for each of the patients we calculated a pairwise distance between sample pairs via the Jensen-Shannon distance (JSD) using the R software package “phyloseq”. For the microarray data, the generation (starting from the raw data) and normalization of the RNA expression values was performed using the robust multi-array algorithm (RMA). All remaining analyses were performed using R software. For in vitro and in vivo analyses: Results for each group were compared using Student t test (for comparisons of two groups) and analysis of variance (for multiple group comparisons). For values that were not normally distributed (as determined by the Kolmogorov-Smirnov test), the Mann–Whitney rank sum test was used. A P value less than 0.05 was deemed statistically significant. All other statistical tests for in vitro and in vivo experiments were performed using GraphPad Prism 7 (GraphPad Software, Inc., San Diego, CA). The multiple hypothesis testing correction of these results was made using the FDR.

For manuscripts utilizing custom algorithms or software that are central to the research but not yet described in published literature, software must be made available to editors/reviewers. We strongly encourage code deposition in a community repository (e.g. GitHub). See the Nature Research guidelines for submitting code & software for further information.

Data

Policy information about availability of data

All manuscripts must include a data availability statement. This statement should provide the following information, where applicable:

- Accession codes, unique identifiers, or web links for publicly available datasets
- A list of figures that have associated raw data
- A description of any restrictions on data availability

The microarray data that support the findings of this study have been deposited in the Gene Expression Omnibus (GEO) data bank, accession code (GSE136031).

Field-specific reporting

Please select the one below that is the best fit for your research. If you are not sure, read the appropriate sections before making your selection.

☒ Life sciences ☐ Behavioural & social sciences ☐ Ecological, evolutionary & environmental sciences

For a reference copy of the document with all sections, see nature.com/documents/nr-reporting-summary-flat.pdf

Life sciences study design

All studies must disclose on these points even when the disclosure is negative.

Sample size	Between 5 and 15 mice were assigned per treatment group, based on the specific experiment; this sample size gave approximately 80% power to detect a 50% change in tumor weight with 95% confidence.
Data exclusions	No data were excluded from analyses
Replication	In all experiments we aimed to verify replication and reproducibility by using 3-4 biologic replicates for gene signatures (microarrays), molecular profiling with RNA and protein (qPCR and western blots), in vitro phenotypes (colony formation and migration assays). For in vivo experiments we confirmed our key findings with different breast cancer models (4T1 and EO771.LMB) and also with several different compounds (HDAC inhibitors) with potent inhibition of HDAC11 (quisinostat, vorinostat, entinostat).
Randomization	Where possible, for all in vivo experiments, post cell injection mice were randomized within cages and randomly distributed to cages prior to labeling their groups. Groups were then assigned at random across the different cages prior to treatment.
Blinding	Our animal studies core performed all treatment of mice in our study. This core is blinded to the expected results, allowing us to reduce any potential biasing from treatment.

Reporting for specific materials, systems and methods

We require information from authors about some types of materials, experimental systems and methods used in many studies. Here, indicate whether each material, system or method listed is relevant to your study. If you are not sure if a list item applies to your research, read the appropriate section before selecting a response.

Materials & experimental systems

n/a	Involved in the study	
☐	☒	Antibodies
☐	☒	Eukaryotic cell lines
☒	☐	Palaeontology
☐	☒	Animals and other organisms
☐	☒	Human research participants
☒	☐	Clinical data

Methods

n/a	Involved in the study	
☒	☐	ChIP-seq
☐	☒	Flow cytometry
☒	☐	MRI-based neuroimaging

Antibodies

Antibodies used	Anti-RRM2 was purchased from Novus Biologicals (1:1000, NBP131661), anti-E2F7 was purchased from Novus (1:1000, NBP1-80266), anti-E2F8 was purchased from Novus (1:1000, NBP152650), anti-vinculin was purchased from Sigma (1:1000, V9131).
Validation	Novus Statement regarding antibodies: Recombinant protein encompassing a sequence within the center region of RRM2. The exact sequence is proprietary. Validated for western blot use in mice and humans. The E2F7 antibody is a rabbit polyclonal antibody against E2F7 and was validated on Western blot. The E2F8 antibody used an immunogen for this product that maps to a region between residue 817 and 867 of human E2F Transcription Factor 8 using the entry NP_078956.2 (GeneID 79733). This is confirmed to bind E2F8 in human and mouse for western blot. The Sigma antibody for vinculin is validated to work in mouse and human for western blotting.

Eukaryotic cell lines

Policy information about cell lines

Cell line source(s)	4T1 cells were obtained from the ATCC and maintained in RPMI containing 10% FBS. EO771.LMB cells were generatred by and obtained directly from Robin Anderson (Peter MacCallum Cancer Center).
Authentication	4T1 cells were fingerprinted by the ATCC, EO771.LMB were authenticated by Dr. Anderson prior to shipping

Mycoplasma contamination	All cells were routinely tested for mycoplasma using a Lonza MycoAlert Detection kit (LT07-418).
Commonly misidentified lines (See ICLAC register)	N/A

Animals and other organisms

Policy information about studies involving animals; ARRIVE guidelines recommended for reporting animal research

Laboratory animals	Adult Balb/c were purchased from Taconic Farms and C57Bl/6 mice were purchased from Jackson Labs.
Wild animals	N/A
Field-collected samples	N/A
Ethics oversight	These animals were cared for according to guidelines set forth by the American Association for Accreditation of Laboratory Animal Care and the U.S. Public Health Service policy on Human Care and Use of Laboratory Animals. All mouse studies were approved and supervised by the University of North Carolina at Chapel Hill Institutional Animal Care and Use Committee.

Note that full information on the approval of the study protocol must also be provided in the manuscript.

Human research participants

Policy information about studies involving human research participants

Population characteristics	From the UNC Breast Cancer Rapid Autopsy Program, all clinical samples were obtained and prepared following informed consent and institutional review board approval for UNC Chapel Hill.
Recruitment	Breast cancer patients from the rapid autopsy program were recruiting in UNC's breast medical oncology clinic.
Ethics oversight	UNC's institutional review board

Note that full information on the approval of the study protocol must also be provided in the manuscript.

Flow Cytometry

Plots

Confirm that:

- ☒ The axis labels state the marker and fluorochrome used (e.g. CD4-FITC).
- ☒ The axis scales are clearly visible. Include numbers along axes only for bottom left plot of group (a 'group' is an analysis of identical markers).
- ☒ All plots are contour plots with outliers or pseudocolor plots.
- ☒ A numerical value for number of cells or percentage (with statistics) is provided.

Methodology

Sample preparation	Fluorescently labeled 4T1 cells were stained with Live/Dead fixable violet dye (Thermo Scientific) for 15 minutes, after which, cells were washed and suspended in FACS buffer (1% bovine serum albumin in PBS containing 0.5 mM EDTA).
Instrument	Cells were analyzed using a Cyan (Beckman Coulter) or Attune NxT (Life Technologies) flow cytometer.
Software	FCS files were analyzed using FlowJo software (version 10; FlowJo LLC).
Cell population abundance	The mCherry-positive population was defined based on fluorescence intensity of 4T1-mCherry cells compared with 4T1 unlabeled cells.
Gating strategy	The starting cell population was gated to include only high FSC/SSC to remove debris, then doublets were excluded by side scatter plot. Live cells were then gated using the Live/Dead Fixable Violet stain. The "dead" cell population was defined using a heat-treated control.

☒ Tick this box to confirm that a figure exemplifying the gating strategy is provided in the Supplementary Information.
